# New LncRNAs in Chronic Hepatitis C progression: from fibrosis to hepatocellular carcinoma

**DOI:** 10.1038/s41598-020-66881-2

**Published:** 2020-06-18

**Authors:** Adriana Camargo Ferrasi, Geysson Javier Fernandez, Rejane Maria Tommasini Grotto, Giovanni Faria Silva, Joao Goncalves, Marina C. Costa, Francisco J. Enguita, Maria Inês de Moura Campos Pardini

**Affiliations:** 1 Department of Internal Medicine, Medical School, Sao Paulo State University (UNESP), Botucatu, SP Brazil; 2 Molecular Biology Laboratory, Blood Transfusion Center, HC-FMB, Sao Paulo State University (UNESP), Botucatu, SP Brazil; 3 Grupo Inmunovirologia, Facultad de Medicina, Universidad de Antioquia, UdeA, Medellín Colombia; 4 Department of Bioprocess and Biotechnology, College of Agricultural Sciences, Sao Paulo State University (UNESP), Botucatu, SP Brazil; 5 iMed-Research Institute of Medicines, Faculdade de Farmácia da Universidade de Lisboa, Lisboa, Portugal; 6 Instituto de Medicina Molecular João Lobo Antunes, Faculdade de Medicina, Universidade de Lisboa, 1649-028 Lisboa, Portugal

**Keywords:** Molecular biology, Oncogenesis

## Abstract

Hepatocellular carcinoma (HCC) is the third leading cause of cancer-related death in the world, and about 80% of the cases are associated with hepatitis B or C. Genetic and epigenetic alterations are accumulated over decades of chronic injury and may affect the functioning of tumor suppressor genes and protooncogenes. Studies have evidenced the role of Long non-coding RNAs (LncRNA) with oncogenic or tumor suppressor activities, suggesting a great potential in the treatment, diagnosis or indicator of prognosis in cancer. In this context, the aim of this study was to evaluate the global expression profile lncRNA in hepatic tissue samples with different stages of fibrosis associated with chronic hepatitis C, HCC and normal liver, in order to identify new lncRNAs that could contribute to study the progression of hepatic fibrosis to HCC associated with chronic hepatitis C. RNA-Seq was performed on Illumina NextSeq platform to identify lncRNAs expressed differently in 15 patients with chronic hepatitis C, three patients with HCC and three normal liver specimens. When the pathological tissues (fibrosis and carcinoma) were compared to normal hepatic tissue, were identified 2, 6 e 34 differentially expressed lncRNAs in moderate fibrosis, advanced fibrosis and HCC, respectively. The carcinoma group had the highest proportion of differentially expressed lncRNA (34) and of these, 29 were exclusive in this type of tissue. A heat map of the deregulated lncRNA revealed different expression patterns along the progression of fibrosis to HCC. The results showed the deregulation of some lncRNA already classified as tumor suppressors in HCC and other cancers, as well as some unpublished lncRNA whose function is unknown. Some of these lncRNAs are dysregulated since the early stages of liver injury in patients with hepatitis C, others overexpressed only in tumor tissue, indicating themselves as candidates of markers of fibrosis progression or tumor, with potential clinical applications in prognosis as well as a therapeutic target. Although there are already studies on lncRNA in hepatocellular carcinoma, this is the first study conducted in samples exclusively of HCV-related liver and HCV HCC.

## Introduction

Hepatocellular carcinoma (HCC) is the sixth most common type of cancer and the third leading cause of cancer-related death in the world^1^. Surgical resection and liver transplantation are the main resources for treatment. However, the results are limited, and the prognosis is poor due to late diagnosis, distant metastases and high risk of postoperative recurrence^2–4^. Due to unspecific or absent symptoms in HCC, early detection requires the bi-annual monitoring of individuals at risk through ultrasound with or without assessment of the alpha-fetoprotein (AFP) biomarker^5^. Environmental and genetic factors are involved in hepatic carcinogenesis. About 80% of the cases are associated with hepatitis B or C^6,7^, mainly due to the development of cirrhosis after years of chronic infection^4^. Despite advances in the diagnosis and treatment of hepatitis C, which effectively reduce the risks for hepatocellular carcinoma^8,9^, these resources are still not widely available to all patients, therefore, hepatocellular carcinoma still represents a public health problem.

HCV chronic infection promotes, in the long term, inflammation, chronic liver injury, fibrosis, dysplasia, and cirrhosis^10^. Hepatic fibrosis is characterized by excess accumulation of extracellular matrix resulting from the healing process of chronic lesions suffered by the liver cells, caused by various pathogenic factors. Over time, the accumulation of excess fibrous connective tissue distorts the normal parenchymal structure of the organ, forming septa and nodules that impair portal blood flow, leading to hypertension and cirrhosis. This tissue context is accompanied by the recruitment of immune cells whose cytokines and growth factors stimulate fibrogenesis. Thus, chronic stimulation of tissue regeneration and the pro-inflammatory environment could somehow, predispose to the development of hepatocellular carcinoma^11,12^. In Western countries, including Brazil, 70–80% of HCC cases are associated with cirrhosis secondary to chronic B or C virus infection^13,14^. Genetic and epigenetic alterations are accumulated over decades of chronic injury and may affect the functioning of tumor suppressor genes and protooncogenes, essential for the control of proliferation, metabolism, adhesion and cell death^15,16^. The lncRNAs are transcribed larger than 200 base pairs, devoid of open read frame (ORF). They are transcribed by RNA polymerase II and are frequently processed and polyadenylated^17^. The expression of these molecules is tissue-specific and varies according to the physiological cell status^18,19^. The lncRNAs could regulate the expression of protein coding genes, both proximally (cis) and distally (trans)^20^, as well as acting at epigenetic^21^ and/or post-transcriptional levels^22^. Studies have demonstrated the role of various lncRNAs with oncogenic or tumor suppressor activities, suggesting a great potential in the treatment, diagnosis or indicator of prognosis in cancer^23–25^. The development of cancer is a multi-step process involving genetic and cellular changes, and advances in molecular techniques, such as next generation sequencing, have shown that genetic alterations precede histological derangements^26–29^ and are accumulated step by step during the malignant transformation of normal cells into cancer cells. This knowledge reinforces the importance of the search for new lncRNAs involved in the carcinogenesis and progression of HCC. In this context, the aim of this study was to evaluate the global expression profile of non-coding long RNAs (lncRNA) in hepatic tissue samples with different stages of fibrosis associated with chronic hepatitis C, hepatocellular carcinoma and normal liver, in order to identify new lncRNAs that could contribute to study the progression of hepatic fibrosis to hepatocellular carcinoma associated with chronic hepatitis C virus infection.

## Materials and methods

### Subject samples and ethics statement

Liver tissue specimens enrolled in the study, were collected in the Viral Hepatitis Outpatient Clinic of Botucatu Medical School, UNESP, Brazil. The case groups consisted of 15 patients with chronic hepatitis C, 3 patients with hepatocellular carcinoma (stage II; AJCC 8th TNM staging system) and the control group was composed for 3 specimens of normal liver. For the case group only were included detectable HCV-RNA cases, no previous hepatitis C treatment (naïve patients) or anti-cancer therapy and known fibrosis stage or clinical diagnosis of cirrhosis by image. The METAVIR^30^ grading was used, considering F0 (absent fibrosis), F1-F2 (moderate fibrosis) and F3-F4 (advanced fibrosis). Liver tissue specimens from patients with hepatitis C were collected by percutaneous biopsy and HCC samples were obtained immediately after surgical resection. This study was approved by the Ethics Committee on Research of Sao Paulo State University, conform to the provisions of the Declaration of Helsinki. All participants of the study signed individual informed consent forms.

### Tissue sample preparation and RNA extraction

Liver tissue samples were stored in RNA later and stored at −80 °C. A sample of 50 mg tissue was triturated using Precellys 24-Dual homogenizer (Bertin Technologies, Rockville, Washington D.C., USA). The total RNA was extracted using TRIzol (Thermo Scientific, USA), according to the manufacturer’s instructions. The RNA was quantitated by spectrophotometry using Qubit RNA Assay Kit in Qubit 2.0 Flurometer (Thermo Scientific, USA). RNA Integrity was ensured by obtaining RNA Integrity Number - RIN > 8 with Agilent 2100 Bioanalyzer (Agilent Technologies, Germany). RNA samples were treated with DNA Free Kit (Thermo Scientific, USA) to remove genomic DNA contamination.

### Library preparation and Illumina sequencing

Each sample was diluted to the same concentration and each cDNA library was prepared from a *pool* of total RNAs from hepatic tissues with the following characteristics: a) Absent fibrosis (F0): RNA pooled of 3 specimens in F0 stage; b) Moderate fibrosis (F1, F2): RNA pooled of 3 specimens in F1 stage and 3 specimens in F2 stage; c) Advanced fibrosis (F3, F4): RNA pooled of 3 specimens in F3 stage and 3 specimens in F4 stage; d) Hepatocellular carcinoma: RNA pooled of 3 specimens of hepatocellular carcinoma; e) Normal liver: 3 non-tumor liver tissue samples and classified as normal after histopathological analysis. Libraries for Illumina sequencing were constructed from 1 µg of total RNA with the TruSeq Stranded Total RNA Sample Preparation Kit v2 (Illumina). The constructed cDNA libraries were qualified by Agilent 2100 Bioanalyzer, quantified by qPCR with KAPA Library Quantification Kit (Kapa Biosystems, Inc.), and sequencing was performed on the NextSeq. 500 System (Illumina) platform using the NextSeq. 500/550 High Output v2 150 cycles (Illumina) sequencing kit.

### Read Alignment and differential gene expression

The data output in fastq file format. Average Phred scores of ≥20 per position were used for alignment. Paired-end reads for RNA were mapped to the human GRCh38 genome build using TopHat2^31^, with this option–mate-inner-dist 200–mate-std-dev 100–no-novel-juncs–min-intron-length 40. Counts for RefSeq genes were obtained using HTSeq^32^ with the default settings and DESeq. 2 (version 1.4)^33^ was used to normalize expression counts. The changes in gene expression were considered statistically significant when |fold change | (FC) ≥ 1.5 and p-values ≤ 0.05. For assess mRNA transcript abundance the reads were converted to reads per Thousand base pairs peak per million mapped reads (RPKM).

### Gene ontology (GO) enrichment analysis

GO enrichment was performed using the ClueGO Cytoscape plugin^34^, using a hypergeometric test with a Benjamini and Hochberg False Discovery Rate correction. A p-value cut-off of 0.05 was used to identify enriched processes.

## Results and Discussion

**Figure 1:**
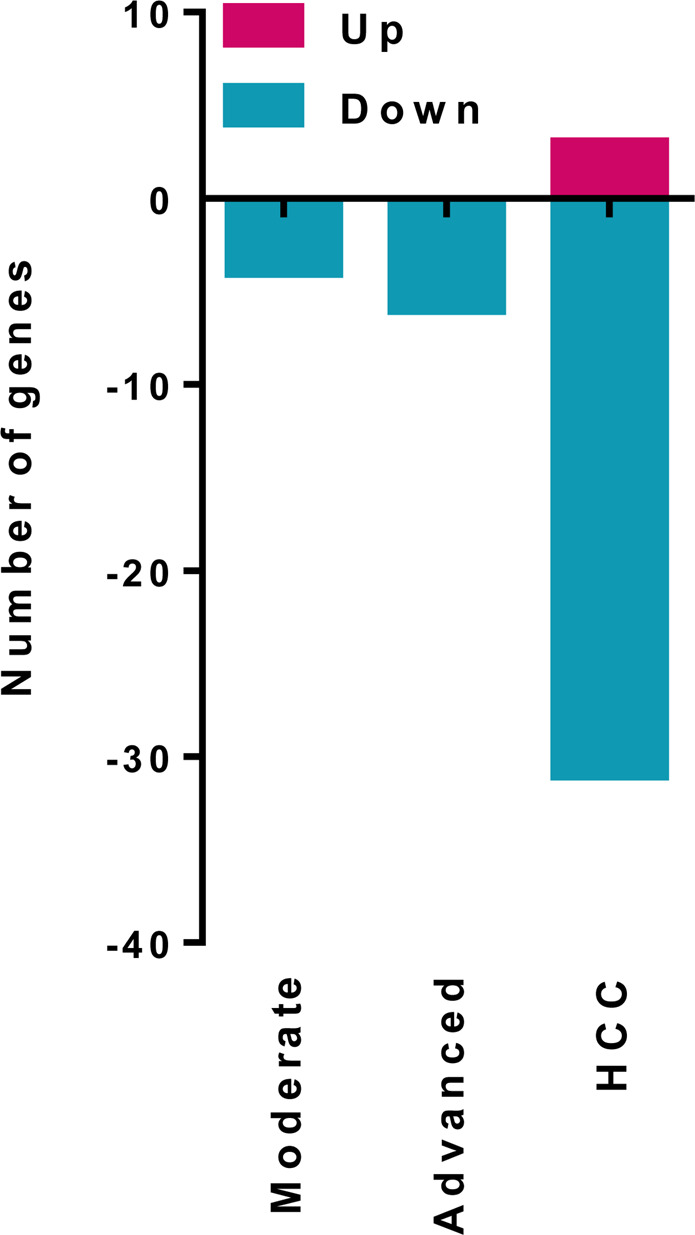
Bar plot representing the total number of up-regulated and down-regulated genes in moderate fibrosis, advanced fibrosis and hepatocellular carcinoma groups.

In this study, we obtained the differential expression profile of lncRNA from hepatic tissues with different stage of fibrosis in chronic hepatitis C, hepatocellular carcinoma and normal tissue by Illumina-based RNA-seq. A total of 36 lncRNA were differentially expressed (p ≤ 0.05 and |fold change | ≥1.5). Table 1 shows the differentially expressed lncRNAs. When the pathological tissues (fibrosis and carcinoma) were compared to normal hepatic tissue, we identified 4, 6 and 34 differentially expressed lncRNAs in moderate fibrosis, advanced fibrosis and hepatocellular carcinoma groups, respectively (Fig. 1). No significant differential expression of lncRNAs was detected in the absent fibrosis group (F0).

**Table 1:** LncRNA differentially expressed (p ≤ 0.05 and fold change ≥1.5) from hepatic tissues with different stage of fibrosis in chronic hepatitis C, hepatocellular carcinoma and normal tissue by Illumina-based RNA-seq.

lncRNA	Name/Description	Class	Cytogenetic location	Differential expression	Phenotype
LINC01748	Long intergenic non-protein coding RNA 1748	antisense	1p32.1	up-regulated	unknown
AL928742.1	New transcript, Uncharacterized	intergenic	14q32.33	up-regulatedin	unknown
MMP2-AS1	MMP2 antisense RNA 1	antisense	16q12.2	up-regulatedin	unknown
HULC	Highly up-regulatedin liver cancer RNA	intergenic	6p24.3	down-regulated	up-regulated in HCC and others tumors
LINC02197	Long intergenic non-protein coding RNA 2197	intergenic	5q13.2	down-regulated	unknown
LINC02535	Long intergenic non-protein coding RNA 2535	intergenic	6q14.3	down-regulated	unknown
LINC00844	Long intergenic non-protein coding RNA 844	intergenic	10q21.1	down-regulated	unknown
AC008549.1	New transcript, Uncharacterized	intergenic	chr.5	down-regulated	unknown
LINC01018	Long intergenic non-protein coding RNA 1018	intergenic	5p15.31	down-regulated	down-regulated in HCC
LINC01093	Long intergenic non-protein coding RNA 1093	intergenic	4q35.1	down-regulated	down-regulated in HCC
LINC02532	Long intergenic non-protein coding RNA 2532	intergenic	6q21	down-regulated	unknown
CTXND1	Long Intergenic Non-Protein Coding RNA 1314	antisense	15q25.1	down-regulated	down-regulated in gastric cancer and hepatoblastoma
AC025271.2	New transcript, Uncharacterized	antisense	15q21.3	down-regulated	unknown
LINC02037	Long intergenic non-protein coding RNA 2037	intergenic	3q29	down-regulated	unknown
AC016044.1	New transcript, Uncharacterized	intergenic	15q21.3	down-regulated	unknown
AC021242.2	New transcript, Uncharacterized	intergenic	8p23.1	down-regulated	unknown
AC010280.3	New transcript, Uncharacterized	intergenic	5q13.1	down-regulated	unknown
LINC01817	Long intergenic non-protein coding RNA 1817	intergenic	2q23.3	down-regulated	unknown
LINC01818	Long intergenic non-protein coding RNA 1818	intergenic	2q23.3	down-regulated	unknown
AL035706.1	New transcript, Uncharacterized	intergenic	1p31.1	down-regulated	unknown
AL139161.1	New transcript, Uncharacterized	intergenic	1q42.3	down-regulated	unknown
LINC02027	Long intergenic non-protein coding RNA 2027	intergenic	3p12.2	down-regulated	down-regulated in HCC
LINC01780	Long intergenic non-protein coding RNA 1780	intergenic	1p12	down-regulated	unknown
LINC02499	Long intergenic non-protein coding RNA 2499	intergenic	4q13.3	down-regulated	unknown
AC105105.2	New transcript, Uncharacterized		18q21.31	down-regulated	unknown
AC015845.2	New transcript, Uncharacterized	intergenic	17q22	down-regulated	unknown
LINC01370	Long intergenic non-protein coding RNA 1370	intergenic	20q12	down-regulated	unknown
AC087521.1	New transcript, Uncharacterized	antisense	11p11.2	down-regulated	unknown
AP001043.1	New transcript, Uncharacterized	intergenic	21q22.2	down-regulated	unknown
AP001043.2	New transcript, Uncharacterized	intergenic	21q22.2	down-regulated	unknown
LINC00313	Long Intergenic Non-Protein Coding RNA 313	intergenic	21q22.3	down-regulated	upregulated in lung and papillary thyroid cancer
AP001781.1	New transcript, Uncharacterized	intergenic	11q23.1	down-regulated	unknown
XIST	X inactive specific transcript	intergenic	Xq13.2	down-regulated	aberrantly expressed in various cancers, including HCC
TSIX	TSIX Transcript, XIST Antisense RNA	intergenic	Xq13.2	down-regulated	HCC
LINC00261	Long Intergenic Non-Protein Coding RNA 261	sense_intronic	20p11.21	down-regulated	down-regulated in HCC and others tumors
AC115619.1	New transcript, Uncharacterized	intergenic	2p24.1	down-regulated	unknown

**Figure 2:**
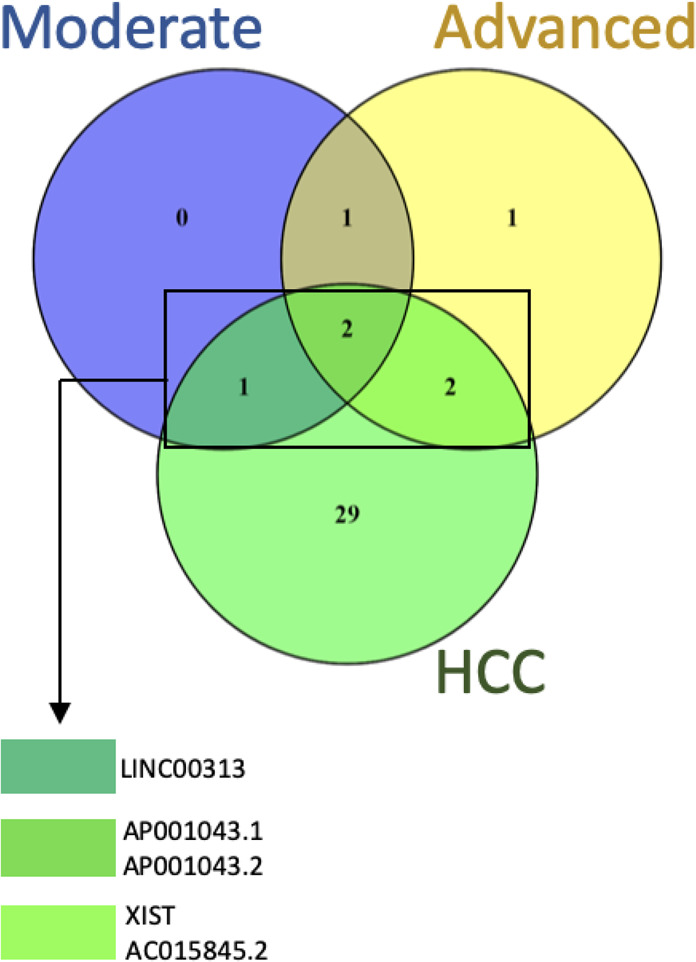
Venn diagram of the differentially expressed lncRNA: The number in each circle represents the amount of differentially expressed genes between the different comparisons: moderate and advanced fibrosis and hepatocellular carcinoma (HCC) groups.

Additionally, we found two lncRNAs (AP001043.1 and AP001043.2) differentially expressed (down-regulated) in common among the different groups studied (Fig. 2). Both are new intergenic transcripts, located in 21q22.2 *locus* and have never been found deregulated in other disorders.

AC015845.2 and XIST are down-regulated in both advanced fibrosis and hepatocellular carcinoma (Fig. 2). AC015845.2 is a new transcript, located in 17q22 *locus* and not yet characterized and has not yet been related to other diseases. XIST (X-inactive specific transcript) is known for its key role in dosage compensation by randomly inactivating one of the X chromosomes in mammalian females during embryonic development^35^. Recently, Yildirim *et al*. (2013) showed that Xist deletion in mice hematopoietic cells induced highly aggressive myeloproliferative neoplasm and myelodysplastic syndrome (mixed MPN/MDS) *in vivo*. Other studies have investigated the relationship between XIST and human cancer including gastric cancer^36,37^, cervical cancer^38^, osteosarcoma^39^, prostate cancer^40^, glioblastoma^41^, breast cancer^42,43^ and hepatocellular carcinoma^44,45^. It is noteworthy that XIST and AC015845.2 transcripts were found down regulated exclusively in cases of advanced fibrosis and hepatocellular carcinoma, suggesting association with advanced disease.

**Figure 3:**
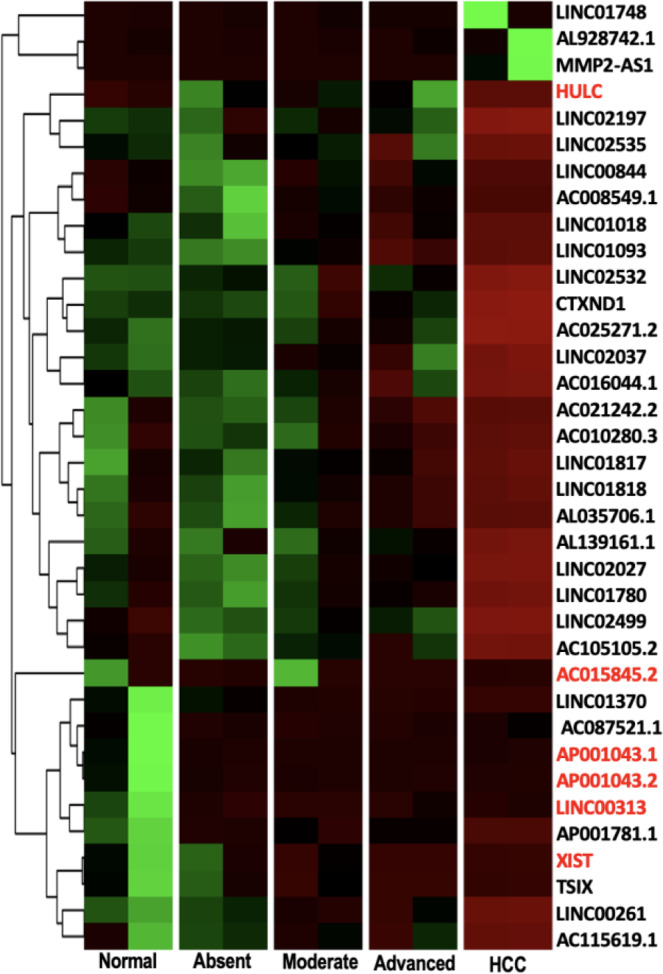
Heatmap of 36 Z-score normalized differentially expressed lncRNA of normal samples, absent fibrosis, moderate fibrosis, advanced fibrosis and hepatocellular carcinoma (HCC) by unsupervised hierarchical clustering analysis. Down-regulated and up-regulated lncRNA are shown in red and green, respectively.

HCC group had the highest proportion of differentially expressed lncRNA (34 lncRNAs) and of these, 29 were exclusive in this type of tissue (Fig. 2). A heatmap of the differentially expressed lncRNA revealed different expression patterns along the progression of fibrosis to hepatocellular carcinoma (Fig. 3). Three lncRNAs were up-regulated in HCC, and 31 were down-regulated. A search for the function of lncRNAs in databases (GENCODE, lncRNAdb, LNCipedia, GeneCards, Ensembl and OMIM) was performed. Fifteen of them are new transcripts and have not yet been characterized. However, some transcripts have already been described as involved in some types of tumors (Table 1), including hepatocellular carcinoma, reinforcing its association with HCC.

HULC (Highly up‐regulated in liver cancer) was originally identified as the most overexpressed long non‐coding RNA in hepatocellular carcinoma^46^. It plays a key role in tumorigenesis, such as promotion of cell survival, proliferation, invasion and angiogenesis^47,48^. Currently, up‐regulation of HULC has been demonstrated in other cancer types, including gastric cancer, pancreatic cancer, osteosarcoma and hepatic metastasis of colorectal cancer, as reviewed by Yu *et al*. (2016)^49^. Interestingly, our results presented here indicate HULC down-regulated in HCC samples (Fig. 3). Although the mechanisms underlying HULC overexpression in many cancer types remain uncertain, studies have suggested a link between HULC overexpression and HBV infection^50^ by HBV X protein (HBx), an oncogenic viral protein involved in HBV pathogenicity, both in HCC and in non-tumor tissues of the liver^51^. HULC levels increases according to tumor grade and aggressiveness, indicating its utility as a prognostic indicator^52^. HULC studies on HCV-related carcinoma are scarce, so the non-agreement of our data with studies published in HBV-related HCC could suggest that HULC dysregulation occurs by a different pathway in HCV-related HCC, stimulating further investigations. In addition, the tumors analyzed here were classified as grade I of Edmondson and Steiner, which report the lower levels of HULC expression.

LINC01018, also called SRHC, was down-regulated in HCC, corroborating the findings of Zheng *et al*.^53^ that found lower levels of expression in HCC samples with a high serum a-fetoprotein level and a low degree of differentiated tumors. In addition, up-regulation of LINC01018 was shown to decrease proliferation while promoting apoptosis of HCC cells^54^. LINC02027 (alias LOC728290) was found down-regulated in HCC, as also observed by Zhang *et al*.^55^. Down-regulation was positively associated with increased serum α-fetoprotein levels and microvascular invasion. Determination of serum α-fetoprotein levels is essential in the clinical diagnosis of liver cancer, suggesting that LINC02027 may be a promising biomarker for HCC. Our data show that lower levels of LINC01018 and LINC02027 can already be observed since advanced fibrosis, showing potential as early markers of HCC.

LINC00261 was down-regulated in moderate fibrosis, advanced fibrosis and hepatocellular carcinoma groups. Recent studies indicate that LINC00261 acts as a tumor suppressor involved in several tumors, such as gastric cancer, lung cancer, choriocarcinoma and HCC^56^. Upregulation LINC00261 significantly inhibited Notch signaling pathway, aberrantly activated in tumors and plays the tumor-promoting role in HCC^56^.

LINC01093 shows significant downregulation in HCC tissues in our study and, recently, He *et al*.^57^ correlated with HCC TNM stage and as a prognostic predictor for HCC patients. Overexpression significantly suppresses HCC cell proliferation and metastasis *in vitro* and *in vivo*. Conversely, its knockdown promotes HCC progression.

Other down-regulated lncRNAs found in our study (CTXND1 and LINC00313) were also associated with other tumors, such as hepatoblastoma, lung, gastric and papillary thyroid cancers (Table 1).

It is noteworthy that three lncRNAs (LINC01748, AL928742.1 and MMp2-AS1) found overexpressed were observed exclusively in the HCC. Both are novel transcripts and studies should be conducted to clarify their cellular function and their role as tumor marker.

**Figure 4:**
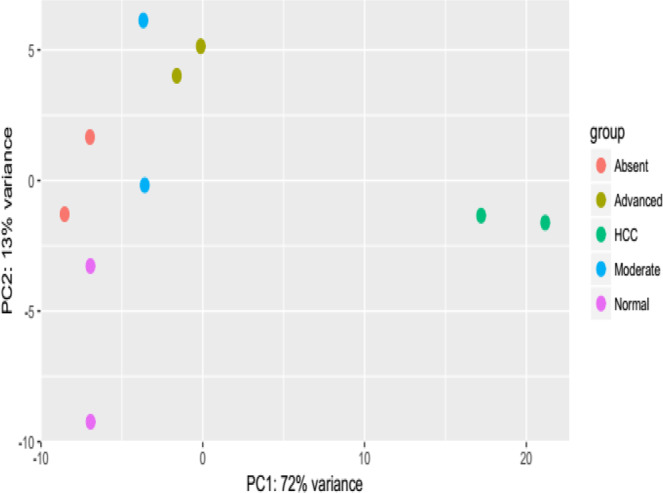
Principal component analysis (PCA) of the lncRNA expression data of normal samples, absent fibrosis, moderate fibrosis, advanced fibrosis and hepatocellular carcinoma (HCC).

The inspection of the lncRNAs through principal component analysis (PCA), revealed a distinct separation of the hepatocellular carcinoma from the other groups (Fig. 4). Moreover, the PCA uncovered a heterogeneity on transcriptome regulation in non-tumor groups, evidenced by the spatial dispersion of the samples in the PCA. This heterogeneity in transcriptome is expected since the progression of the hepatic lesion is a stochastic process and each patient develop variable hepatic condition until the carcinogenesis.

Although there are already studies on lncRNA in hepatocellular carcinoma, this is the first study conducted in samples exclusively of HCV-related liver and HCV HCC.

These findings are limited to the population studied due to the small sample size, however, the observed data are interesting and should encourage further studies with samples from different populations.

The results of the present study showed the deregulation of some lncRNA already classified as tumor suppressors in HCC and other cancers, as well as some unpublished lncRNA whose function is unknown. Some of these lncRNAs are dysregulated since the early stages of liver injury in patients with hepatitis C, others overexpressed only in tumor tissue, indicating potential clinical applications in prognosis as well as a therapeutic target.
